# Competing risks of cancer mortality and cardiovascular events in individuals with multimorbidity

**DOI:** 10.15256/joc.2014.4.41

**Published:** 2014-08-18

**Authors:** Elizabeth A. Bayliss, Liza M. Reifler, Chan Zeng, Deanna B. McQuillan, Jennifer L. Ellis, John F. Steiner

**Affiliations:** ^1^Institute for Health Research, Kaiser Permanente Colorado, Denver, CO, USA; ^2^Department of Family Medicine, University of Colorado School of Medicine, Aurora, CO, USA

**Keywords:** Comorbidity, cancer, cardiovascular disease, shared decision-making, epidemiologic methods

## Abstract

**Background:**

Cancer patients with cardiovascular and other comorbidities are at concurrent risk of multiple adverse outcomes. However, most treatment decisions are guided by evidence from single-outcome models, which may be misleading for multimorbid patients.

**Objective:**

We assessed the interacting effects of cancer, cardiovascular, and other morbidity burdens on the competing outcomes of cancer mortality, serious cardiovascular events, and other-cause mortality.

**Design:**

We analyzed a cohort of 6,500 adults with initial cancer diagnosis between 2001 and 2008, SEER 5-year survival ≥26%, and a range of cardiovascular risk factors. We estimated the cumulative incidence of cancer mortality, a serious cardiovascular event (myocardial infarction, coronary revascularization, or cardiovascular mortality), and other-cause mortality over 5 years, and identified factors associated with the competing risks of each outcome using cause-specific Cox proportional hazard models.

**Results:**

Following cancer diagnosis, there were 996 (15.3%) cancer deaths, 328 (5.1%) serious cardiovascular events, and 542 (8.3%) deaths from other causes. In all, 4,634 (71.3%) cohort members had none of these outcomes. Although cancer prognosis had the greatest effect, cardiovascular and other morbidity also independently increased the hazard of each outcome. The effect of cancer prognosis on outcome was greatest in year 1, and the effect of other morbidity was greater in individuals with better cancer prognoses.

**Conclusion:**

In multimorbid oncology populations, comorbidities interact to affect the competing risk of different outcomes. Quantifying these risks may provide persons with cancer plus cardiovascular and other comorbidities more accurate information for shared decision-making than risks calculated from single-outcome models. Journal of Comorbidity 2014;4:29–36

## Introduction

Over 70% of cancer patients have comorbidities, and at least 30% have two or more coexisting conditions [1–4]. Comorbid cardiovascular disease is a particular concern as prevalence estimates suggest that (depending on age) 11–38% of persons with cancer also have established cardiovascular disease [4, 5]. The majority of postmenopausal women diagnosed with breast cancer have equal or greater long-term risks for cardiovascular outcomes than for cancer mortality, and face lower overall survival with pre-existing cardiovascular disease [6, 7]. Similarly, the majority of men diagnosed with prostate cancer are at greater risk of other-cause mortality than cancer mortality [8]. In addition, many cancer treatments have cardiotoxic side effects, thus increasing cancer patients’ risk of future adverse cardiovascular outcomes [9, 10].

**Figure 1:**
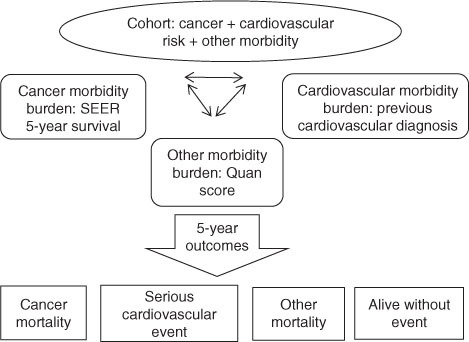
Potential interactions and outcomes in a multimorbid incident cancer cohort. SEER, Survival, Epidemiology, and End Results.

Comanagement of comorbid cancer and cardiovascular disease is currently limited by an evidence base that does not consider the interactions between cancer prognosis, cardiovascular morbidity burden, and other morbidities. Current practice recommendations for these comorbidities are primarily informed by disease-specific treatment guidelines, descriptive studies of cardiovascular risk factor control in cancer survivors, limited predictive models, or expert panel recommendations [11–21]. However, risk calculations derived from models that examine cancer or cardiovascular outcomes separately may be not be relevant for multimorbid individuals because these models misestimate the effect of predictive factors: cardiovascular and other morbidity burdens may affect the risk of cancer mortality; while, at the same time, cancer prognosis may affect the risk of adverse cardiovascular outcomes or other-cause mortality [15–21] (Figure 1). Accurate information on interacting factors that predict the *concurrent* risks of cancer, cardiovascular, and other outcomes can help prioritize treatment strategies and inform shared decision-making in the face of incident cancer diagnoses [18, 22].

## Objective

We proposed to examine the evidence gap of quantifying the competing risks of adverse cancer and cardiovascular outcomes in cancer patients. We assessed the interacting effects of cancer prognosis, cardiovascular morbidity, and non-cancer, non-cardiovascular (‘other’) morbidity on the competing outcomes of cancer mortality, serious cardiovascular events, and other-cause mortality. Our study population consisted of individuals with incident cancer diagnoses with >25% predicted 5-year survival, a range of cardiac risk factors, and other chronic conditions. We hypothesized that while cancer and cardiovascular morbidity burdens would be strong predictors of cancer mortality and serious cardiovascular events, respectively, different types of morbidity would interact to differentially affect the risk of all outcomes.

## Materials and methods

### Study design, population, and setting

We conducted a retrospective cohort study of the outcomes of cancer mortality, serious cardiovascular events, and ‘other cause’ mortality as a function of cancer prognosis, cardiovascular morbidity burden, and non-cancer, non-cardiovascular morbidity (Figure 1). The cohort consisted of adult members of the Kaiser Permanente Colorado (KPCO), an integrated, not-for-profit health maintenance organization, with an initial cancer diagnosis, between January 2001 and December 2008. Eligible individuals were enrolled in the KPCO for at least 1 year prior to diagnosis, had a calculated SEER 9 (Survival, Epidemiology, and End Results), 5-year cancer survival probability of 26% or greater, based on cancer site and stage at diagnosis (http://seer.cancer.gov/statfacts/), and a range of cardiovascular risk factors. Cardiovascular risk factors considered for cohort eligibility included: taking cholesterol-lowering (‘statin’) medication and/or having low-density lipoprotein cholesterol not at goal within 2 years prior to cancer diagnosis; a family history of coronary artery disease (CAD), premature CAD, or stroke; or diagnoses of hypertension, CAD, abdominal aortic aneurism, congestive heart failure, stroke, diabetes, peripheral vascular disease, or chronic kidney disease prior to cancer diagnosis [23]. Supplementary Table 1 contains the ICD-9 diagnosis code and data source information for cardiovascular risk factors. Cancer diagnoses were identified through the KPCO tumor registry, which are case-reviewed and meet Colorado State cancer registry requirements.

The three competing outcomes of interest were: (1) cancer mortality; (2) a composite cardiovascular outcome of ‘serious cardiovascular events’ comprised of myocardial infarction, coronary revascularization, or death from a cardiovascular cause; and (3) mortality from other causes during all available person-days of follow-up time after cancer diagnosis. Ascertainment of death from cancer, serious cardiovascular event, or other causes was based on death data from the state of Colorado and the health plan. Independent variables of interest were: (1) Cancer morbidity as calculated by SEER 5-year survival prognosis; (2) significant cardiovascular morbidity indicated by known previous cardiovascular diagnoses (abdominal aortic aneurism, peripheral vascular disease, stroke, or CAD); and (3) ‘other morbidity’ as calculated by a modified Charlson Comorbidity Index that excluded cardiovascular and cancer diagnoses (m-CCI). We included covariates of age, sex, low socioeconomic status based on aggregate census data, statin use, diabetes, smoking status, and systolic blood pressure. All data were obtained from health claims, the electronic medical record, pharmacy databases, or the KPCO tumor registry.

The investigation was approved by the KPCO Institutional Review Board.

## Analysis

To estimate the overall incidence of cancer mortality, serious cardiovascular events, and death from other causes in the competing risk survival context, we estimated unadjusted cumulative incidence functions. Subgroup incidence curves were also generated by prognosis and type of malignancy. We then produced separate, cause-specific Cox proportional hazard (CSH) models to examine factors associated with each of the primary outcomes using the first event occurring after cancer diagnosis [24, 25]. In the first model, cancer deaths were treated as a failure event, and all other events were treated as a censored event; in the second model, serious cardiovascular events were treated as a failure event, and all other events as censored; and in the third model, deaths from other causes were treated as a failure event, and all other events as censored. For each model, individuals were censored if they experienced any of the competing events, disenrolled from the health plan, or reached the end date of the observation period without an event (December 31, 2010). To confirm that the three outcomes were best modeled as competing outcomes rather than considered as a single outcome, we developed a single survival model and compared the fit of the single-outcome model with the three CSH models with regard to the same set of predictive variables. We used the likelihood ratio test to compare the sum of the fit for the three individual CSH models to the survival model that did not distinguish between the three types of event.

All variables in the original analytic data set were based on established clinical risk factors for the selected outcomes. We tested and retained covariate forms that generated the best model fit, and explored interactions among independent variables of interest and collinearity among covariates. Three variables that were not significantly associated with any of the outcomes in multivariable models and were dropped from final models were baseline diabetes, smoking status, and systolic blood pressure. Martingale residuals, Schoenfeld residuals, and time–covariate interactions were used to assess the proportional hazards assumption for covariates in each model [26]. Significant interactions with time were modeled in periods that achieved the best fit. Wald χ^2^-tests were conducted to test whether parameter estimates in the CSH models for each outcome significantly differed from each other. Because individuals who experienced a non-fatal serious cardiovascular event were potentially still at risk of cancer or other mortality, we conducted sensitivity analyses using CSH models in which we allowed individuals who had a non-fatal cardiovascular event and later died of cancer or other causes to be ‘counted’ as having these second outcomes. Statistical Analysis System (SAS) 9.2 (Cary, NC, USA) was used for all analyses.

## Results

**Figure 2:**
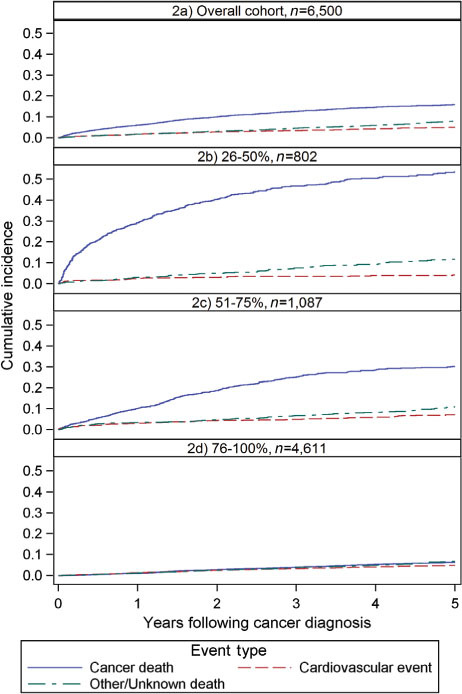
Cumulative incidence of outcomes: overall and by SEER (Survival, Epidemiology, and End Results) prognosis.

The study cohort consisted of 6,500 members who met the inclusion criteria. Table 1 lists the baseline characteristics of this cohort. Over a median follow-up period of 3.7 years (interquartile range 2.2–5.9 years) following cancer diagnosis, there were 996 (15.3%) cancer deaths, 328 (5.1%) serious cardiovascular events, 542 (8.3%) deaths from other causes, and 4,634 (71.3%) cohort members with none of these outcomes. Accounting for censoring and competing events, the 5-year cumulative incidence was 16.0% for cancer deaths, 5.1% for serious cardiovascular events, and 8.1% for other cause mortality (Figure 2).

Cumulative incidence functions stratified by cancer prognosis (Figure 2) illustrate a greater 5-year incidence of cancer mortality among those with a worse cancer prognosis, and increasingly comparable risks for the three outcomes with improving SEER prognosis. The subcohort with 26–50% 5-year survival experienced 53.5% cancer mortality, 4.2% serious cardiovascular events, and 11.8% other mortality; those with 51–75% 5-year survival experienced 30.3% cancer mortality, 7.1% serious cardiovascular events, and 11.0% other mortality; and those with 76–100% 5-year survival experienced 6.2% cancer mortality, 4.8% serious cardiovascular events, and 6.8% other mortality.

Results of the CSH models for the effects of independent variables on cancer mortality, serious cardiovascular events, and other mortality are shown in Table 2. Table 2 also illustrates significant differences *between* the effects of independent variables on each outcome. The CSH models for the separate outcomes had a significant reduction in error compared with the model with combined outcomes (likelihood ratio χ^2^ statistic=328.32, *p*≤0.001), supporting the assumption that these three outcomes should be modeled as competing risks rather than as a single outcome in this population.

A worse cancer prognosis was associated with a significant increase in risk for all outcomes, with the greatest effect on cancer mortality. This association was strongest in the year following diagnosis. In subsequent years, SEER prognosis remained a significant predictor of cancer and other mortality, but not of serious cardiovascular events. Cardiovascular morbidity burden was also significantly associated with all outcomes, and this effect was significantly greater on the outcomes of serious cardiovascular events and other mortality than on the outcome of cancer mortality. ‘Other’ morbidity burden increased the risk of all three outcomes in the subpopulation with the best cancer prognosis, and increased the risk of cancer mortality and other mortality in those with SEER prognoses of 51–75%. Interactions between cancer and cardiovascular morbidity burdens, and between statin use and previous cardiovascular diagnoses were not significant in any CSH models, and were omitted from the final models.

Cause-specific hazard ratios for all outcomes as a function of cardiovascular morbidity and other morbidity within SEER cancer prognosis strata are presented in Table 3. These illustrate, by cancer prognosis, the interacting effects of cardiovascular other morbidity burden on the selected outcomes.

Of cohort members, 104 (1.6%) first had a serious cardiovascular event and later died of cancer or other cause. In sensitivity analyses, allowing for both events to occur in these individuals did not change the hazard ratios for any of the outcome events. Thus, the original CSH models with time to first event are reported.

## Discussion

In the absence of accurate and specific information, clinicians and cancer patients may either underestimate or overestimate risks of adverse outcomes from cancer and other morbidities [27–34]. In this analysis, studying competing risks more accurately estimates the effect of three different types of morbidity on the outcomes of cancer mortality, serious cardiovascular events, and other-cause mortality than would single-outcome models. Although previous competing hazard analyses have demonstrated decreased cancer mortality and increased other-cause mortality with increased morbidity burden in cancer patients, we are unaware of any studies that quantify the competing risk of serious cardiovascular events in the face of incident cancer [8, 35].

In our study population, cancer prognosis, cardiovascular morbidity, and other morbidity significantly and independently predicted all outcomes. The strength of these associations was affected by time and by cancer prognosis. This evidence, coupled with an appreciation of patient goals and priorities, can help guide individualized decision-making for multimorbid oncology patients. Specifically, for cancer patients with cardiovascular risk factors, predictors of serious cardiovascular events can inform the management of cardiovascular risk reduction as well as decisions about cancer treatment.

Two examples based on our results illustrate such decision-making. For a patient with a 40% SEER probability of 5-year cancer survival, a history of a myocardial infarction alone would not significantly increase her risk of any of the outcomes over a 5-year follow-up period (Table 3). Although her relative risk of a serious cardiovascular event is more strongly affected by her cardiac history, her estimated absolute risk remains greatest for cancer mortality (Figure 2b) which may guide her treatment decisions. Alternatively, for a different patient with a new diagnosis of a good prognosis cancer (SEER probability of 5-year survival >75%), a history of cardiovascular disease plus two other chronic conditions would increase his relative risk of cancer mortality by 2.6 times, but increase by 3.7 times his risk of a subsequent serious cardiovascular event and increase by 4.6 times his risk of other mortality (Table 3). His baseline 5-year risk is low and comparable for each of these events (Figure 2d), suggesting that he should pay particular attention to ongoing chronic disease management throughout his cancer treatment.

In addition to informing individual decision-making, our results can inform population-level cardiovascular risk management in cancer patients. Our study population was defined in part by cardiovascular risk, and the majority of cohort members in this investigation experienced neither cancer mortality, nor a serious cardiovascular event, nor other-cause mortality during the 5 years after diagnosis. This demonstrates the importance of appropriate cardiovascular risk management in the face of incident cancer – ideally through sharing care between primary care and oncology [36]. Although there is conflicting information about the quality of chronic disease management after cancer diagnosis, shared care results in better management of comorbid conditions and more preventive services [12, 13, 36–41].

There are several limitations to this study. The cohort reflects the population of an integrated care system. Since all cohort members in this analysis had some degree of cardiovascular risk, we likely understate the impact of pre-existing cardiovascular diagnoses on outcomes relative to a broader cancer population. We did not have information on cancer treatment, and considerations of cancer prognosis and overall morbidity may well have influenced treatment choices and subsequent outcomes. We did not focus on one type of cancer, but intentionally selected a heterogeneous patient population since comorbid cardiovascular risk can occur across many malignancies. Future longitudinal analyses of larger cohorts with specific malignancies that permit age stratification and long-term follow-up will provide more detail on competing risks of cardiovascular and other outcomes in specific clinical settings [25, 42]. Finally, we examined only three outcomes; information on the risk of other important adverse outcomes, as well as contextual factors and personal preferences will be important to guide decision-making for individuals with cancer and comorbid medical conditions.

In spite of these limitations, this investigation has two significant strengths. First, we believe this is the first investigation to quantify the concurrent risks of cancer mortality, serious cardiovascular events, and other mortality in the face of incident cancer. This is an important addition because many persons with incident cancer have some degree of cardiovascular risk. And second, this study further demonstrates the importance of using competing hazard analyses when studying outcomes in multimorbid populations. Single-outcome models can tell us that morbidity burden and cancer prognosis are independent predictors of cancer mortality; but competing hazards analyses more accurately illustrate the relative effects of these predictors on cancer mortality, serious cardiovascular events, and other mortality over the same time period.

The majority of current clinical prediction tools remain based on single-outcome models. Using data from electronic health records and other sources, competing risk models that incorporate interacting predictors of multiple outcomes (such as the ones illustrated here) should be an important component of clinical decision support. Applications such as the Cancer Survival Query System (which uses competing risk methodology to predict cancer versus other mortality for prostate and colorectal cancer based on age and morbidity burden) are an important step in this direction [35]. Other investigations that build on these approaches will greatly improve decision-making in oncology care for multimorbid patients.

## Conclusions

In multimorbid oncology populations, survivorship care begins at cancer diagnosis [3, 7]. Comorbidities interact to affect the risks of multiple different outcomes, and accurate calculation of these competing risks in the context of morbidity burden, personal priorities, and care needs can help optimize shared decision-making. Given the potential prevalence of cardiovascular risk factors among cancer patients, risk of cardiovascular events may be an important outcome category for many patients. Such information can inform ongoing management of cardiovascular risk factors throughout cancer diagnosis and treatment.

## Figures and Tables

**Table 1:** Characteristics of the study population.

Variable	All *n* (%) or mean (SD) (*N*=6,500)
Outcome	
Cancer death	996 (15.3%)
CV event	328 (5.1%)
Death (other cause)	542 (8.3%)
Censored^a^ (<1 year)	241 (3.7%)
Censored^a^ (1–5 years)	2,499 (38.5%)
Censored^a^ (>5 years)	1,894 (29.1%)
Year of diagnosis	
2001	659 (10.1%)
2002	726 (11.2%)
2003	789 (12.1%)
2004	713 (11.0%)
2005	815 (12.5%)
2006	878 (13.5%)
2007	998 (15.4%)
2008	922 (14.2%)
Age at diagnosis	66.7 (11.5)
Sex	
Male	3,225 (49.6%)
Female	3,275 (50.4%)
Low SES	882 (13.6%)
Previous CV diagnosis	1,875 (28.8%)
Cancer site	
Bladder	431 (6.6%)
Breast	1,451 (22.3%)
Colon	788 (12.1%)
Endometrium	216 (3.3%)
Head and neck	241 (3.7%)
Kidney and renal pelvis	207 (3.2%)
Lung	184 (2.8%)
Non-Hodgkin lymphoma	397 (6.1%)
Prostate	1,349 (20.8%)
Skin melanoma	437 (6.7%)
Other	799 (12.3%)
On statin at diagnosis	2,136 (32.9%)
SEER 5-year survival	
26–50%	802 (12.3%)
51–75%	1,087 (16.7%)
76–100%	4,611 (70.9%)
Other morbidity^b^ (number of conditions)	
0	3,209 (49.4%)
1	1,822 (28.0%)
≥2	1,469 (22.6%)
Diabetes at baseline	1,243 (19.1%)
Baseline smoking status (ever/never)	1,005 (15.5%)
Baseline systolic BP	132.2 (18.8)

BP, blood pressure; CV, cardiovascular; SD, standard deviation; SEER, Survival, Epidemiology, and End Results; SES, socioeconomic status.

^a^Censored refers to disenrollment from the health plan, or reaching the end date of the observation period without an event (December 31, 2010).

^b^Modified Charlson Comorbidity Index (excludes cancer and cardiovascular morbidity).

**Table 2:** Adjusted cause-specific hazards of outcomes [hazard ratios (95% CI); overall *n*=6,500].^a,e^

Parameter	Cancer mortality^b^(*n*=996)	CV event^b^(*n*=328)	Other mortality^b^(*n*=542)
Age at diagnosis	**1.04 (1.04–1.05)**^d^	**1.05 (1.04–1.06)**	**1.07 (1.06–1.08)**^d^
Female sex	**1.14 (1.00–1.29)**^c^	**0.74 (0.59–0.93)**^c^	0.94 (0.94–1.12)
Low SES	**1.34 (1.15–1.57)**	1.18 (0.88–1.57)	1.10 (0.88–1.38)
Previous CV diagnosis (yes/no)	**1.34 (1.17–1.55)**^c,d^	**2.20 (1.72–2.82)**^c^	**1.79 (1.48–2.16)**^d^
SEER 5-year survival prognosis (referent group 76–100%)			
26–50%, year 1	**32.45 (23.37–45.07)**^c,d^	**2.35 (1.32–4.18)**^c^	**3.05 (1.80–5.17)**^d^
26–50%, years 2–5	**9.49 (7.57–11.89)**^c,d^	0.99 (0.51–1.92)^c^	**3.15 (2.23–4.44)**^d^
51–75%, year 1	**8.87 (6.20–12.70)**^c,d^	**2.26 (1.41–3.61)**^c^	**2.53 (1.59–4.05)**^d^
51–75%, years 2–5	**4.76 (3.83–5.92)**^c,d^	1.37 (0.93–2.02)^c^	**1.60 (1.19–2.15)**^d^
Two or more chronic conditions^f^			
SEER 5-year survival 26–50%	1.20 (0.98–1.48)	0.88 (0.41–1.87)	1.49 (0.96–2.32)
SEER 5-year survival 51–75%	**1.49 (1.17–1.89)**	1.17 (0.72–1.92)	**2.01 (1.38–2.92)**
SEER 5-year survival 76–100%	**1.89 (1.46–2.44)**	**1.72 (1.29–2.30)**	**2.62 (2.09–3.30)**

CI, confidence interval; CV, cardiovascular; SEER, Survival, Epidemiology, and End Results; SES, socioeconomic status.

^a^Significant associations between independent variables and individual outcomes are shown in **bold.** Significant differences across outcomes are footnoted.

^b^For each model, individuals were censored if they experienced any of the competing outcomes, disenrolled from the health plan, or reached the end date of the observation period event free. All models are adjusted for statin use at baseline.

^c^Significant difference between cancer mortality and cardiovascular event at *p*<0.05.

^d^Significant difference between cancer mortality and other mortality at *p*<0.05

^e^Significant difference between cardiovascular event and other mortality at *p*<0.05.

^f^Modified Charlson Comorbidity Index (excludes cancer and cardiovascular morbidity).

**Table 3:** Cause-specific hazards of outcomes as a function of morbidity stratified by SEER prognosis. Data are hazard ratios (95% CI).^a^

SEER 5-year survival group	Group	Cancer death HR	CV event HR	Other death HR
26–50% (*n*=802)	Previous CV condition	1.08 (0.81–1.42)	2.04 (0.82–5.08)	1.47 (0.81–2.68)
	Other morbidity alone	0.98 (0.74–1.31)	0.51 (0.11–2.35)	1.25 (0.66–2.35)
	Both CVD and morbidity	1.64 (1.23–2.17)	2.12 (0.77–5.80)	2.44 (1.31–4.56)
51–75% (*n*=1,087)	Previous CV condition	1.53 (1.13–2.06)	1.94 (1.06–3.57)	1.91 (1.15–3.17)
	Other morbidity alone	1.82 (1.29–2.56)	1.15 (0.46–2.84)	2.49 (1.39–4.47)
	Both CVD and morbidity	1.91 (1.38–2.65)	2.48 (1.28–4.79)	3.43 (2.05–5.73)
76–100% (*n*=4,611)	Previous CV condition	1.66 (1.20–2.31)	2.60 (1.82–3.70)	2.28 (1.70–3.07)
	Other morbidity alone	2.16 (1.52–3.09)	2.22 (1.43–3.45)	3.37 (2.46–4.63)
	Both CVD and morbidity	2.55 (1.76–3.68)	3.69 (2.49–5.49)	4.56 (3.33–6.25)

CI, confidence interval; CV, cardiovascular; HR, hazard ratio; SEER, Survival, Epidemiology, and End Results; SES, socioeconomic status.

^a^Adjusted for age, sex, low SES (yes/no), and statin use at baseline.
